# Prediction of DNA-binding proteins from relational features

**DOI:** 10.1186/1477-5956-10-66

**Published:** 2012-11-12

**Authors:** Andrea Szabóová, Ondřej Kuželka, Filip Železný, Jakub Tolar

**Affiliations:** 1 Czech Technical University, Prague, Czech Republic; 2 University of Minnesota, Minneapolis, USA

**Keywords:** DNA-binding propensity prediction, DNA-binding proteins, Relational machine learning

## Abstract

**Background:**

The process of protein-DNA binding has an essential role in the biological processing of genetic information. We use relational machine learning to predict DNA-binding propensity of proteins from their structures. Automatically discovered structural features are able to capture some characteristic spatial configurations of amino acids in proteins.

**Results:**

Prediction based only on structural relational features already achieves competitive results to existing methods based on physicochemical properties on several protein datasets. Predictive performance is further improved when structural features are combined with physicochemical features. Moreover, the structural features provide some insights not revealed by physicochemical features. Our method is able to detect common spatial substructures. We demonstrate this in experiments with zinc finger proteins.

**Conclusions:**

We introduced a novel approach for DNA-binding propensity prediction using relational machine learning which could potentially be used also for protein function prediction in general.

## Background

The process of protein-DNA interaction has been an important subject of recent computational-biology research, however, it has not been completely understood yet. DNA-binding proteins have a vital role in the biological processing of genetic information like DNA transcription, replication, maintenance and the regulation of gene expression. Several computational approaches have recently been proposed for the prediction of DNA-binding function from protein structure. In this paper we are interested in prediction of DNA-binding propensity of proteins using their structural information and physicochemical properties. This approach is in contrast with some of the most recent methods which are based on similarity of proteins, for example structural alignment or threading-based methods [1-3] or methods exploiting information about evolutionary conservation of amino acids in proteins [4]. In general, methods exploiting evolutionary information can be more accurate than the approaches aiming to infer binding propensity purely from physicochemical or structural protein properties. On the other hand, the main advantage of the approaches not using evolutionary information is that they do not rely on the existence of homologous proteins and also they may provide interpretable patterns describing the binding principles.

In one of the pioneering works on the prediction of DNA-binding propensity, Stawiski et al. [5] investigated the structural and sequence properties of large, positively charged electrostatic patches on DNA-binding protein surfaces. They used a neural network with 12 features such as molecular weight per residue, patch size, percent *α*-helix in patch, average surface area per residue, number of residues with hydrogen-bonding capacity, percent of patch and cleft overlap, number of lysine and polar isosteres in Lys_*off*_patches, and percent of conserved positive and aromatic residues in the patch. Ahmad and Sarai [6] trained a neural network based on the net charge, the electric dipole and quadrupole moments of the protein. The combination of charge and dipole moment performed best, while information about the quadrupole moment improved the accuracy only slightly. They found out that DNA-binding proteins have significantly higher net positive charges and electric moments than other proteins. Bhardwaj et al. [7] examined the sizes of positively charged patches on the surface of DNA-binding proteins. They trained a support vector machine classifier using the protein’s overall charge and its overall and surface amino acid composition. Szilágyi and Skolnick [8] created a logistic regression classifier based on the amino acid composition, the asymmetry of the spatial distribution of specific residues and the dipole moment of the protein. Patel et al. [9] used an artificial neural network to discriminate DNA-binding proteins from non-DNA binding proteins using amino-acid sequential information. For each amino acid sequence they created a set of 62 sequence features. Nimrod et al. [4] presented a random forest classifier for identifying DNA-binding proteins among proteins with known 3D structures. First, their method detects clusters of evolutionarily conserved regions on the surface of proteins using the PatchFinder algorithm. Next, a classifier is trained using features like the electrostatic potential, cluster-based amino acid conservation patterns, the secondary structure content of the patches and features of the whole protein, including all the features used by Szilágyi and Skolnick [8].

In the present work, we use an automatic feature construction method based on relational machine learning to discover structural patterns capturing spatial configuration of amino acids in proteins. Numbers of occurrences of each discovered pattern in a protein become attributes of the protein, which are then used by a machine learning algorithm to predict the DNA-binding propensity of the protein. We combine two categories of features to predict the DNA-binding propensity of proteins. The first category contains physicochemical features which enabled Szilágyi and Skolnick’s method [8] to achieve state-of-the-art predictive accuracies. The second category contains structural features representing the discovered spatial patterns in protein structures. Using predictive classifiers based on these features we obtain accuracies competitive with existing physicochemical based methods on several datasets of proteins. Moreover, our method is able to detect conserved spatial substructures, which we demonstrate in experiments with zinc finger proteins.

Nassif et al. [10] previously used a relational learning based approach in a similar context, in particular to classify hexose-binding proteins. The main differences of our approach from the method of Nassif et al. [10] are as follows. First, the fast relational learning algorithm [11] that we use enables us to produce features by inspecting much larger structures (up to tens of thousands of entries in a learning example) than those considered in the work of Nassif et al. [10] using the standard learning system Aleph. Second, our structural features acquire values equal to the number of occurrences of the corresponding spatial pattern, whereas Nassif et al. [10] only distinguished the presence of a pattern in a learning example from its absence. Our preliminary results [12] indicated that occurrence-counting indeed substantially lifts predictive accuracy. Lastly, the approach of Nassif et al. [10] resulted in classifiers that are more easily interpretable than state-of-the-art classifiers and comparable in predictive accuracy. Here we maintain the interpretability advantage and achieve accuracies competitive to the state-of-the-art predictive accuracies both by a purely structural approach (without the physicochemical features) and also through the combination of structural and physicochemical features.

## Materials and methods

### Data

DNA-binding proteins are proteins that are composed of DNA-binding domains. A DNA-binding domain is an independently folded protein domain that contains at least one motif that recognizes double- or single-stranded DNA. We worked with the following datasets in our experiments: 

• PD138 - dataset of 138 DNA-binding protein structures in complex with DNA,

• UD54 - dataset of 54 DNA-binding protein structures in unbound conformation,

• BD54 - dataset of 54 DNA-binding protein structures in DNA-bound conformation corresponding to the set UD54

• APO104 - dataset of 104 DNA-binding protein structures in unbound conformation,

• ZF - dataset of 33 Zinc Finger protein structures in complex with DNA,

• NB110 - dataset of 110 non-DNA-binding protein structures,

• NB843 - dataset of 843 non-DNA-binding protein structures.

Dataset PD138 was created using the Nucleic Acid Database (NDB) by Szilágyi and Skolnick [8] - it contains a set of DNA-binding proteins in complex with DNA strands with a maximum pairwise sequence identity of 35% between any two sequences.

Both the protein and the DNA can alter their conformation during the process of binding. This conformational change can involve small changes in side-chain location, and also local refolding, in case of the proteins. Predicting DNA-binding propensity from a structural model of a protein makes sense if the available structure is not a protein-DNA complex, i.e. it does not contain a bound nucleic acid molecule. In order to find out how the results would change according to the conformation before and after binding, we used two other datasets (UD54, BD54). BD54 contains bound conformations of DNA-binding proteins, i.e. DNA-protein complexes. UD54 contains the same sequences in their unbound, free conformation. These datasets were also obtained from Szilágyi and Skolnick [8].

Another set of DNA-binding protein structures (APO104) determined in the absence of DNA was obtained from Gao et al. [2].

Thirty-three examples of Cys_2_His_2_ZF-DNA complexes were sourced from Siggers et al. [13]. Their structural description was obtained from the Protein Data Bank.

Rost and Sander constructed a dataset (RS126) for secondary structure prediction. Ahmad & Sarai [6] removed the proteins related to DNA binding from it, thus getting a final dataset of non-DNA-binding proteins. As our negative dataset (NB110) we used this set of non-DNA-binding proteins.

We also used an extended dataset (NB843) by Nimrod et al. [4]. This dataset contains additional 733 structures of non-DNA-binding proteins. The additional structures were gathered using the PISCES server. Entries in this list include crystal structures with a resolution better than 3.0Å. The sequence identity between each pair of sequences is smaller than 25%.

From the structural description of each protein we extracted the list of all contained residues with information on their type and the list of pairwise spatial distances among all residues. As for the physicochemical features, we followed Szilágyi and Skolnick’s work [8] and extracted features indicating the respective proportions of the Arg, Lys, Asp, Ala and Gly residues, the spatial asymmetry of Arg, Gly, Asn and Ser, and the dipole moment of the protein.

### Method

Our method exploits techniques of relational machine learning [14] in conjunction with state-of-the-art attribute-value learning algorithms [15]. Very briefly, our method can be viewed as proceeding in three steps. It starts with PDB files, which is a widely used format for proteins. Then it creates a relational representation of the proteins (*step 1*). After that it tries to extract meaningful relational patterns from the relational structures describing proteins and uses them to create an approximate attribute-value representation of the proteins (*step 2*) which is then used for learning attribute-value classifiers (*step 3*).

Although the field of attribute-value machine learning is more mature than the field of relational machine learning, attribute-value learning algorithms, such as decision trees or support vector machines, suffer from the limitation that they can deal only with data which is in the form of data tuples (such as real-valued or boolean vectors) of fixed length. Attribute-value learning algorithms face problems when dealing with data in a more structured form, for example spatial structures of proteins. On the other hand, relational learning algorithms can directly learn from data expressed as relational structures such as graphs or the logic-based form which we adopt and explain below. Spatial structures of proteins, which is what we are interested in, can be represented very naturally within the relational-learning framework.

*Propositionalization*[16] is a general strategy which combines advantages of attribute-value learning algorithms (usually higher accuracy) and relational learning algorithms (ability to handle structured examples). In propositionalization, one tries to convert a relational learning problem to an attribute-value learning problem by *transforming* the original relational representation to an (approximate) attribute-value representation, i.e. to representation where learning examples are represented as vectors of fixed size, and then to train an attribute-value classifier for such data. Thus, roughly speaking, propositionalization corresponds to steps *2* and *3* of our method.

The representation of examples that we use is rooted in the field of inductive logic programming [14] which is a sub-field of relational learning. However, for brevity, we mostly avoid the whole logical machinery usually used in inductive logic programming and we speak instead (rather informally) about relational structures instead of first-order formulas and logical interpretations. A *literal* is an expression of the form *literalName*(*A*_1_,…,*A*_*k*_) where *A*_1_,…,*A*_*k*_ are variables or constants. We use the convention from logic programming that variables start with an upper-case letter. For example *residue*(*A*,*his*) or *distance*(*AB*,10.0Å) are literals and *A, B* are variables whereas *his* and 10Å are constants. An example is simply a set of literals none of which contains a variable. For instance

$$\begin{array}{ll} \;e_{1}= & \text{residue}(a,\text{glu}),\text{residue}(b,\text{cys}),\text{distance}(a,b,4.0\text{Å}),\\ & \text{distance}(b,a,4.0\text{Å})\; \end{array}$$

is an example describing a dipeptide.

Besides examples, we also need *patterns*. A pattern is a set of literals which, unlike examples, may contain variables. An example of a pattern is

$$p_{1}=\text{residue}(A,X),\text{distance}(A,B,10.0\text{Å}),\text{residue}(B,\text{glu})$$

A pattern *p* is said to cover^a^ an example *e* when we are able to find a substitution *θ* to variables of *p* such that *pθ *⊆* e*. For example the pattern *p*_1 _covers the example *e*_1 _because *p*_1_*θ *⊆* e* for substitution *θ *= {*A*/*bB*/*a*}. We are not interested only whether a pattern *p* covers a given example *e* but also how many *covering substitutions* there are, i.e. how many substitutions *θ *such that *pθ *⊆* e *there are. We call the number of covering substitutions of a pattern *p* its value. Although counting the number of covering substitutions is not very common in ordinary propositionalization approaches, it makes perfect sense for the problem of predicting DNA-binding propensity of proteins, since ability to bind DNA is often connected with count or proportion of atom-groups with certain properties (e.g. charged residues [17]).

In the experiments we used a representation of proteins that consisted of literals representing types of the residues and literals representing pair-wise distances between the residues up to 10Å. These distances were computed from alpha-carbon coordinates obtained from PDB^b^. We also restricted the shapes of possible patterns by insisting that the patterns have to be tree-like. Despite these simplifications, some of the examples contained, in the end, tens of thousands literals which would be very challenging for common relational learning systems such as Aleph^c^, not to mention that these systems do not allow computing numbers of covering substitutions. Therefore we customized the pattern search algorithm [11] which is more appropriate for problems of this size due to its pruning mechanisms and strong structural language bias (it constructs only tree-like patterns). This pattern search algorithm prunes pattern space using two measures: *redundancy* (described by Kuželka et al. [11]) and *minimum frequency* which is a minimum number of examples that must be covered by a pattern. An example of a tree-like pattern is res(A,arg), res(B,arg), res(C,lys), dist(A,B,10.0), dist(A,C,10.0). This pattern assumes the presence of two Arginines – A and B – and one Lysine – C. The distance between the Arginines is 10Å, the distance between the Arginine A and the Lysine C is also 10Å. A pattern like this can be used as a feature, counting the number of occurrences of this particular spatial configuration of amino acids in proteins.

The generated patterns were used for classification using six state-of-the-art attribute-value learning algorithms listed in Table 1. We used implementation of these learning algorithms present in the WEKA [18] open-source machine learning software. We also combined the patterns constructed automatically by the relational pattern search algorithm with numerical features devised by Szilágyi and Skolnick [8].

**Table 1:** Learning algorithms

**Classifier**	**Category**	**References**
Linear support vector machine	kernel	[19]
Support vector machines with RBF kernel	kernel	[19]
Simple logistic regression	regression/ensemble	[20]
L_2_-regularized logistic regression	regression	[21]
Ada-boost (with decision stamps)	ensemble	[22]
Random forest	ensemble	[23]

State-of-the-art attribute-value learning algorithms used for classification.

Parameters of the classifiers were tuned using internal cross-validation. When performing cross-validation, the set of patterns was created separately for each train-test split corresponding to iterations of cross-validation procedure. The number of trees for random forest and the number of iterations for Ada-boost was selected from the set {10,20,50,100,200,500,1000}. The complexity parameter *c* for linear support vector machine and for support vector machine with RBF kernel was selected from the set {1,10,10^2^,10^3^,10^4^,10^5^,10^6^}. The regularization parameter of *L*_2_-regularized logistic regression was selected from the set {10^−3^,10^−2^,10^−1^,1,10,10^2^,10^3^}. The minimum frequency of features on one of the classes was 0.7.

We used a different methodology for experiments with datasets PD138/NB843, because the size of the dataset required a sampling-based approach to feature construction rather than exhaustive search. Therefore, we followed an approach in which patterns were constructed on several randomly selected subsets of data and then evaluated on the complete dataset. The number of random samples was set to 10, the number of proteins in the samples from each class was set to 20. The minimum frequency for each sample was set to 1.

## Results and discussion

We experimented with several datasets to evaluate the predictive accuracy and also the interpretability of our approach. We compared classifiers based on structural patterns discovered by our method (SF) with classifiers based on 10 physicochemical features (PF) identified as most predictive by Szilágyi and Skolnick’s method [8]. We also trained classifiers based on both structural features and physicochemical features (PSF). For each experiment we estimated predictive accuracy and the area under the ROC curve (AUC) by 10-fold cross-validation. Lastly, we inspected the most informative structural patterns in order to evaluate interpretability of these patterns. We assessed the informativeness by the *χ*^2 ^criterion [24].

We performed five sets of experiments with datasets of DNA-binding proteins - PD138, UD54, BD54, APO104 and ZF - each one as a set of positive examples and dataset of non-DNA-binding proteins NB110 - as a set of negative examples. We obtained about 1400 structural patterns for datasets PD138/NB110, approximately 1500 structural patterns for datasets UD54/NB110, about 2400 structural patterns for datasets BD54/NB110, about 2800 structural patterns for datasets APO104/NB110 and approximately 6000 structural patterns for datasets ZF/NB110. Accuracies and areas under the ROC curve (AUC) obtained on the respective datasets by stratified 10-fold cross validation using physicochemical features (PF), structural pattern features (SF) and combination of both of them (PSF) are shown in Table 2. The results for the method based on physicochemical features (PF) differs slightly from the results reported by Szilágyi and Skolnick [8], because we used 10-fold cross-validation whereas Szilágyi and Skolnick used leave-one-out cross-validation.

**Table 2:** Results

		***Accuracy***			***AUC***	
**PD138 vs. NB110**	***PF***	***SF***	***PSF***	***PF***	***SF***	***PSF***
Simple logistic regression	**83.4 (1)**	82.2 (2)	80.7 (3)	0.91 (2)	0.90 (3)	**0.94** (1)
L_2_-regularized log. regression	81.4 (3)	83.5 (2)	**85.5 (1)**	**0.92 (1)**	0.91 (2)	0.91 (2)
SVM with radial basis kernel	81.8 (2)	79.9 (3)	**85.1 (1)**	0.92 (2)	0.90 (3)	**0.93 (1)**
Linear SVM	81.4 (3)	83.6 (2)	**83.9 (1)**	0.92 (2)	0.89 (3)	**0.93 (1)**
Ada-boost w. decision stamps	80.6 (2)	78.6 (3)	**81.4 (1)**	**0.90 (1)**	**0.90 (1)**	**0.90 (1)**
Random forest	81.8 (3)	**83.5 (1)**	82.3 (2)	0.90 (3)	0.91 (2)	**0.93 (1)**
**Average ranking**	2.33	2.17	**1.5**	1.83	2.33	**1.17**
**UD54 vs. NB110**	***PF***	***SF***	***PSF***	***PF***	***SF***	***PSF***
Simple logistic regression	81.0 (3)	**86.0 (1)**	82.8 (2)	**0.91 (1)**	0.89 (2)	0.89 (2)
L_2_-regularized log. regression	82.2 (3)	82.4 (2)	**84.1 (1)**	0.89 (3)	**0.91 (1)**	0.90 (2)
SVM with radial basis kernel	81.0 (2)	**84.0 (1)**	80.4 (3)	**0.92 (1)**	0.88 (3)	0.91 (2)
Linear SVM	81.7 (2)	**82.4 (1)**	**82.4 (1)**	0.90 (2)	**0.91 (1)**	0.87 (3)
Ada-boost w. decision stamps	76.2 (3)	78.0 (2)	**79.3 (1)**	0.88 (3)	0.89 (2)	**0.90 (1)**
Random forest	78.6 (3)	**79.3 (1)**	79.2 (2)	0.88 (3)	0.89 (2)	**0.90 (1)**
**Average ranking**	2.67	**1.34**	1.67	2.17	**1.67**	2
**BD54 vs. NB110**	***PF***	***SF***	***PSF***	***PF***	***SF***	***PSF***
Simple logistic regression	80 (3)	80.5 (2)	**81.8 (1)**	**0.91 (1)**	0.85 (2)	**0.91 (1)**
L_2_-regularized log. regres	**83.1 (1)**	81.9 (2)	81.7 (3)	**0.92 (1)**	0.88 (3)	0.91 (2)
SVM with radial basis kernel	82.5 (2)	82.5 (2)	**83.6 (1)**	**0.91 (1)**	0.90 (2)	0.90 (2)
Linear SVM	81.4 (3)	82.3 (2)	**82.9 (1)**	0.93 (2)	0.90 (3)	**0.94 (1)**
Ada-boost w. decision stamps	**84.2 (1)**	73.8 (3)	79.8 (2)	**0.91 (1)**	0.88 (2)	0.88 (2)
Random forest	**82.4 (1)**	75.0 (3)	79.4 (2)	0.89 (2)	0.89 (2)	**0.91 (1)**
**Average ranking**	1.83	2.33	**1.67**	**1.33**	2.33	1.5
**APO104 vs. NB110**	***PF***	***SF***	***PSF***	***PF***	***SF***	***PSF***
Simple logistic regression	80.7 (3)	**85.0 (1)**	80.8 (2)	0.89 (3)	**0.92 (1)**	0.91 (2)
L_2_-regularized log. regression	82.6 (3)	**84.5 (1)**	83.1 (2)	0.90 (2)	**0.91 (1)**	**0.91 (1)**
SVM with radial basis kernel	79.4 (3)	83.2 (2)	**84.1 (1)**	0.88 (3)	0.90 (2)	**0.91 (1)**
Linear SVM	79.4 (3)	**84.5 (1)**	84.1 (2)	0.89 (2)	0.89 (2)	**0.92 (1)**
Ada-boost w. decision stamps	77.6 (3)	78.1 (2)	**79.1 (1)**	0.87 (2)	0.87 (2)	**0.89 (1)**
Random forest	**81.7 (1)**	78.5 (3)	79.4 (2)	0.88 (2)	0.87 (3)	**0.89 (1)**
**Average ranking**	2.67	**1.67**	**1.67**	2.33	1.83	**1.17**
**ZF vs. NB110**	***PF***	***SF***	***PSF***	***PF***	***SF***	***PSF***
Simple logistic regression	95.1 (3)	**98.7 (1)**	97.2 (2)	0.99 (2)	**1.0 (1)**	**1.0 (1)**
L_2_-regularized log. regres	95.9 (3)	99.3 (2)	**100 (1)**	0.99 (2)	**1.0 (1)**	**1.0 (1)**
SVM with radial basis kernel	95.8 (3)	**99.3 (1)**	98.6 (2)	0.99 (2)	**1.0 (1)**	**1.0 (1)**
Linear SVM	81.4 (3)	**99.3 (1)**	97.8 (2)	**1.0 (1)**	**1.0 (1)**	**1.0 (1)**
Ada-boost w. decision stamps	95.9 (3)	99.3 (2)	**100 (1)**	0.98 (2)	**1.0 (1)**	**1.0 (1)**
Random forest	96.5 (3)	**97.9 (1)**	97.2 (2)	0.99 (2)	**1.0 (1)**	**1.0 (1)**
**Average ranking**	3	**1.33**	1.67	1.83	**1**	**1**

Predictive accuracies and areas under the ROC curve (*AUC*) on 5 classification benchmarks achieved by 6 machine learning algorithms using physicochemical features (*PF*) as proposed by Szilágyi and Skolnick [8], structural features (SF) automatically constructed by our algorithm, and the combination of both feature sets (*PSF*).

We computed average rankings (over several machine learning algorithms) for accuracies and AUCs. The average ranking (over several machine learning algorithms) of classifiers based on structural features (SF) was best on datasets UD54/NB110, APO104/NB110 (tie with PSF) and ZF/NB110 for accuracies and on datasets UD54/NB110 and ZF/NB110 in terms of AUC. The average ranking of classifiers based on combination of structural and physicochemical features (PSF) was highest on datasets PD138/NB110, APO104/NB110 (tie with SF) and BD54/NB110 for accuracies and on datasets PD138/NB110, APO104/NB110 and ZF/NB110 (tie with SF) in terms of AUC. Classifiers based on physicochemical features (PF) obtained highest ranking only for AUCs on dataset BD54/NB110.

We made an additional experiment with datasets PD138/NB843 in order to be able to compare our method with the method of Nimrod et al. [4]. In this experiment we used only the random forest classifier which was also used by Nimrod et al. On this dataset Nimrod et al. obtained AUC 0.9. We obtained AUC 0.84 with the method of Szilágyi and Skolnick, 0.82 with the method based on structural features and 0.82 with the method based on the combination of structural and physicochemical features. It is important to note that unlike the method of Nimrod et al. our method does not rely on information about evolutionary conservation.

In order to find out whether our method did not just capture the consensus patterns of particular protein folds, we performed an experiment in which we made use of the division of DNA-binding proteins (of the dataset PD138) into seven protein groups. Our method was always applied on sets of proteins consisting of all but one protein group, then the obtained classifiers were tested on this excluded group. The resulting accuracies of linear SVM classifier on the excluded groups were reasonably high with the exception of the enzyme group. The enzyme group turned out to be more difficult for DNA-binding prediction also in previous works [5,8].

The performed experiments allow us to evaluate usability of the relational learning approach for prediction of DNA-binding propensity as well as its usability for discovery of interesting spatial patterns in proteins. Results of our experiments suggest that the method is suitable for both of the tasks. Here, we also discuss the factors influencing predictive performance and biological relevancy of discovered structural patterns.

### DNA-binding proteins in general

We made several sets of experiments for DNA-binding proteins in general (datasets PD138, UD54, BD54, APO104). The method based on purely structural features (SF) and the method based on the combination of structural and physicochemical features (PSF) achieved higher predictive accuracies than the method based purely on physicochemical features (PF) - features introduced by Szilágyi and Skolnick [8]. The only exception was in case of the dataset BD54/NB110, where the method based on purely physicochemical features performed better than the method based on purely structural features. The results were not as definite in the case of AUC as in the case of predictive accuracy. The method based on structural features turned out to be better than the method based on physicochemical features on two datasets. Interestingly, these two datasets contain DNA-binding proteins in their unbound conformations. The method based on the combination of structural and physicochemical features was better than the method based on purely physicochemical features on three datasets.

It may seem counter-intuitive that in some of the experiments, physicochemical features (PF) or structural features (SF) outperformed the combined feature set (PSF). However, this is a rather natural manifestation of the overfitting effect; expansion of the feature set may indeed be detrimental especially with small data sets [15].

It is interesting to compare the results for the datasets UD54 and BD54. Dataset UD54 contains DNA-binding proteins in unbound conformation, dataset BD54 contains the same DNA-binding proteins, but in bound conformation with DNA. Whereas the highest predictive accuracies and best AUCs were obtained by the method based on structural features on dataset UD54, this method performed worst on dataset BD54. Interestingly, the number of frequent structural patterns was significantly higher for dataset BD54 (approximately 2400 structural patterns) than for the dataset UD54 (approximately 1500 structural patterns). This suggests that conformational changes after DNA-binding give rise to greater variability of spatial arrangements of some amino acid groups. Moreover, conformational changes may be responsible for increase of spatial asymmetry of some amino acids or protein’s dipole moment. This can explain the better performance of the method based on physicochemical features on the dataset BD54 (recall that these features were selected by experimenting on DNA-binding proteins in bound conformation with DNA by Szilágyi and Skolnick [8]). Also note that prediction of DNA-binding propensity from unbound conformations is more important for practical applications.

We examined the best discovered patterns in detail. For each split of the dataset PD138 induced by 10-fold cross-validation we selected the ten most informative structural patterns according to the *χ*^2 ^criterion. Table 3 shows the number of occurrences of the ten best patterns. There are four structural patterns which are present in all ten folds. The first is res(A), residue(A,arg). This pattern counts the number of Arginines in the protein. It is known that the Arginine plays an important role in the DNA binding process. For now, we are interested in structural patterns. Since this pattern included no spatial information relating to other amino acids, we decided to analyse just the remaining three patterns.

**Table 3:** The most informative patterns for PD138

	**Structural Pattern**	**N**
1	res(A,arg)	10
2	**res(A,arg), res(B,lys), dist(A,B,4.0)**	10
3	**res(A,arg), res(B,arg), res(C,lys), dist(A,B,10.0), dist(A,C,10.0)**	10
4	**res(A,arg), res(B,arg), dist(A,B,6.0)**	10
5	res(A,arg), res(B,lys), dist(A,B,6.0)	9
6	res(A,ile), res(B,arg), res(C,arg), dist(A,B,6.0), dist(A,C,10.0)	7
7	res(A,leu), res(B,glu), res(C,arg), dist(A,B,10.0), dist(A,C,6.0)	7
8	res(A,lys), res(B,arg), dist(A,B,10.0)	7
9	res(A,arg), res(B,arg), res(C,leu), dist(A,B,10.0), dist(A,C,6.0)	7
10	res(A,arg), res(B,arg), dist(A,B,10.0)	6

The ten most informative structural patterns according to the *χ*^2^criterion for the dataset PD138. **N**is the number of folds, for which the actual pattern was one of the ten best patterns.

We inspected how structural patterns are reflected in protein’s primary structure. First, we examined whether amino acids matched by a pattern occur in a preferred order in the proteins’ sequences. We calculated the distribution of permutations of the amino acids matched by the first analysed structural pattern res(A,arg), res(B,lys), dist(A,B,4.0). The distribution of permutations on positive dataset was almost identical. Next, we were looking for relative positions of these amino acids in the sequences of DNA-binding proteins. Mostly the amino acids were situated next to each other in the proteins’ sequences for both permutations of amino acids: [arg,lys] and [lys,arg], i.e. on positions n and n+1. We also obtained occurrences of this pattern, where the amino acids were on positions n and n+3 for permutation [arg, lys].

The next analysed structural pattern was res(A,arg), res(B,arg), res(C,lys), dist(A,B,10.0), dist(A,C,10.0). There were no prevailing permutations for this structural pattern and also no prevailing local arrangements of amino acids in sequence. It would be hard to express this pattern using only primary structure information, unlike in the case of the previous pattern.

The third analysed structural pattern was res(A,arg), res(B,arg), dist(A,B,6.0). The most frequent relative positions of the amino acids were [n, n+2], [n, n+3], [n, n+4], where the first relative positions were approximately two times more frequent than the other two.

### Zinc finger proteins

Zinc finger proteins are one of the most common DNA-binding proteins in eukaryotic transcription factors. Several studies [25-32] have tried to determine the DNA recognition by these proteins. The sequence of three fingers of the protein Zif268, which served as the prototype for understanding DNA recognition by this family of proteins, is shown with the cysteines and histidines involved in zinc coordination indicated in *bold* font in Table 4 (reproduced from Wolfe et al. [32]). *Filledsquares* below the sequences indicate the position of the conserved hydrophobic residues. *Filledcircles* and *stars* indicate residue positions that are involved in phosphate and base contacts (respectively) in most of the fingers. We evaluated relevance of the discovered structural patterns matching them to observations in the paper of Wolfe et al. [32].

**Table 4:** Annotated sequence of three fingers of Zif268


															-1	1	2	3	4	5	6	7	8	9						
Finger 1	P	Y	A	**C**	P	V	E	S	**C**	D	R	R	F	S	R	S	D	E	L	T	R	**H**	I	R	I	**H**	T	G	Q	K
Finger 2	P	F	Q	**C**	R	I	-	-	**C**	M	R	N	F	S	R	S	D	H	L	T	T	**H**	I	R	T	**H**	T	G	E	K
Finger 3	P	F	A	**C**	D	I	-	-	**C**	G	R	K	F	A	R	S	D	E	R	K	R	**H**	T	K	I	**H**	L	R	Q	K
		■									∙		■		⋆		⋆	⋆	■		⋆	∙								∙
				**0**	1	2	3	4	**5**	6	7	8	9	10	11	12	13	14	15	16	**17**	**18**	19	20	21	**22**				
P1				**C**					**C**												**R**	**H**				**H**				
P2				**C**																	**R**	**H**				**H**				
P3				**C**																	**R**	**H**				**H**				

The sequence of the three fingers of Zif268 is shown with the cysteines and histidines involved in zinc coordination indicated in *bold* font. *Filledsquares* below the sequences indicate the position of the conserved hydrophobic residues. *Filledcircles* and *stars* indicate residue positions that are involved in phosphate and base contacts (respectively) in most of the fingers.

We made predictive classification experiments on dataset of zinc finger proteins (ZF). The best results, in terms of accuracy and AUC, were obtained by the method based on structural features. However, here the results were influenced by the fact that the zinc finger proteins were highly homologous. Therefore, we were more interested in the question whether the structural patterns were able to discover some basic characteristic of DNA-binding process shared by zinc finger proteins.

**Figure 1:**
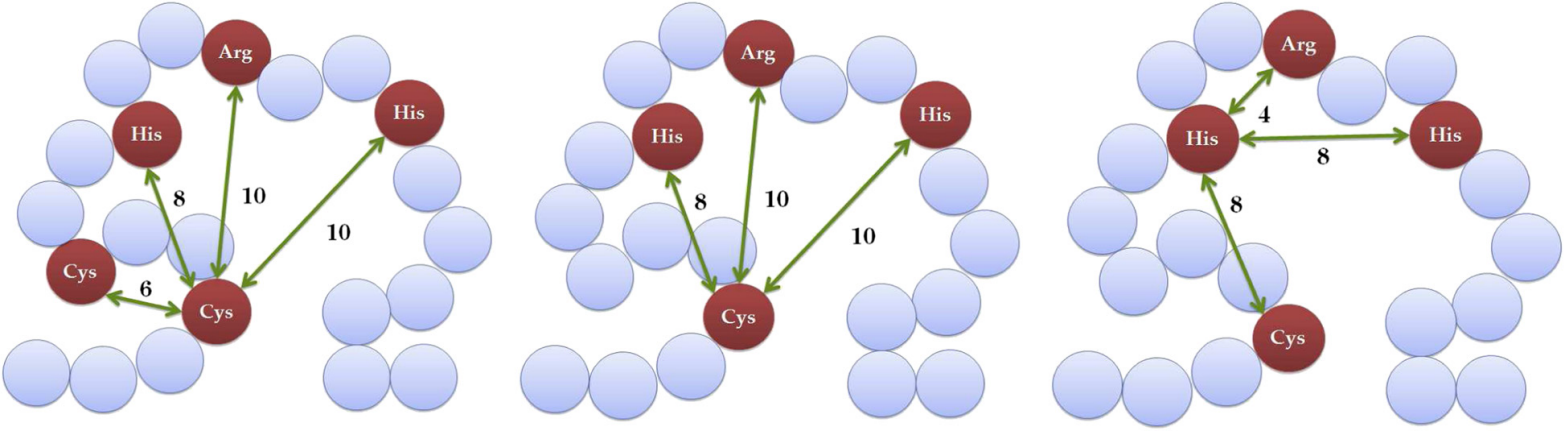
**Structural patterns for Zinc Fingers.** Most informative structural patterns according to the *χ*^2 ^criterion for the data set of Zinc Fingers (edges not to scale).

**Figure 2:**
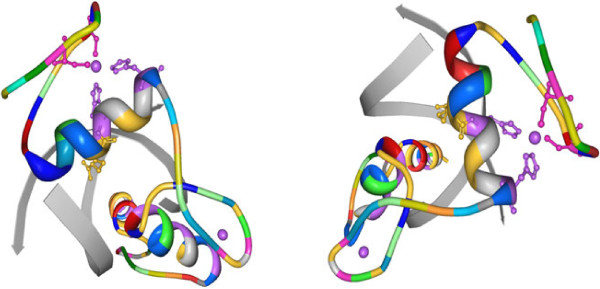
**The most informative structural pattern for Zinc Fingers.** Example proteins (1A1F and 1AAY) containing one discovered pattern shown for the Zinc-finger proteins’ dataset using the protein viewer software [33]. Residues assumed by the pattern are indicated in the following way: CYS - pink, HIS - violet, ARG - yellow.

We inspected the best discovered patterns. We selected the ten most informative structural patterns according to the *χ*^2 ^criterion, following the same procedure as for the DNA-binding proteins in general. Table 5 shows the number of occurrences of the ten best patterns. There were three structural patterns present in all of the dataset splits. We show them in Figures 1 and 2.

**Table 5:** The most informative patterns for ZF

	**Structural pattern**	**N**
1	**res(A,cys), res(B,cys), res(C,his), res(D,his), res(E,arg), dist(A,B,6.0), dist(A,C,8.0), dist(A,D,10.0), dist(A,E,10.0)**	10
2	**res(A,cys), res(B,his), res(C,his), res(D,arg), dist(A,B,8.0), dist(A,C,10.0), dist(A,D,10.0)**	10
3	**res(A,his), res(B,his), res(C,cys), res(D,arg), dist(A,B,8.0), dist(A,C,8.0), dist(A,D,4.0)**	10
4	res(A,cys), res(B,his), res(C,his), res(D,phe), dist(A,B,8.0), dist(A,C,10.0), dist(A,D,8.0)	9
5	res(A,his), res(B,cys), res(C,his), res(D,arg), dist(A,B,10.0), dist(A,C,8.0), dist(A,D,6.0)	9
6	res(A,his), res(B,cys), res(C,his), res(D,arg), dist(A,B,10.0), dist(A,C,8.0), dist(A,D,4.0)	8
7	res(A,cys), res(B,his), res(C,his), dist(A,B,8.0), dist(A,C,10.0)	8
8	res(A,cys), res(B,cys), res(C,his), res(D,his), res(E,phe), dist(A,B,6.0), dist(A,C,8.0), dist(A,D,10.0), dist(A,E,8.0)	8
9	res(A,his), res(B,cys), res(C,his), res(D,phe), res(E,cys), dist(A,B,10.0), dist(A,C,8.0), dist(A,D,10.0), dist(A,E,8.0)	5
10	res(A,his), res(B,cys), res(C,arg), res(D,his), dist(A,B,10.0), dist(A,C,10.0), dist(A,D,8.0)	3

The ten most informative structural patterns according to the *χ*^2 ^criterion for the dataset of Zinc Finger proteins. **N** is the number of folds, for which the actual pattern was one of the ten best patterns.

We calculated the distribution of permutations of the amino acids matched by the first analysed structural pattern res(A,cys), res(B,cys), res(C,his), res(D,his), res(E,arg), dist(A,B,6.0), dist(A,C,8.0), dist(A,D,10.0), dist(A,E,10.0). The most frequent permutation was [cys, cys, arg, his, his]. We looked for the relative positions of these amino acids in zinc finger proteins’ sequences. The most frequently occurring relative positions were: [n, n+5, n+17, n+18, n+22]. We compared this result with the observation described in the paper of Wolfe et al. [32] (reproduced in Table 4). This discovered structural pattern exactly matched the positions of some of the amino acids which are supposed to be directly involved in DNA-binding. In case of the second structural pattern res(A,cys), res(B,his), res(C,his), res(D,arg), dist(A,B,8.0), dist(A,C,10.0), dist(A,D,10.0) and the third structural pattern res(A,his), res(B,his), res(C,cys), res(D,arg), dist(A,B,8.0), dist(A,C,8.0), dist(A,D,4.0) the most frequent permutation was [cys, arg, his, his] and the resulting relative positions were [n, n+17, n+18, n+22]. Table 4 indicates that these two patterns (P2 and P3) cover the first pattern (P1).

While, as already commented, the discovered patterns matched the positions of some of the amino acids supposed to be directly involved in DNA-binding, they in fact do not capture specific properties of DNA-binding process but rather a consensus amino acid pattern known to be present in Cys_2_His_2_ zinc fingers^d^[32]. One could be concerned whether the patterns discovered for DNA-binding proteins in general (datasets PD138, UD54, BD54, APO104) just captured conserved consensus patterns of different folds as well. However, this was not the case, because every discovered pattern was contained in at least 70% of DNA-binding proteins (recall that minimum frequency 0.7 was used for feature construction). In order to assure validity of this claim we performed an additional experiment in which the relational learning model was always constructed for proteins from all but one protein group and then tested on this excluded group (see section*Evaluation of binding motif independence*). Nevertheless, these observations indicate that caution should be exercised when applying our relational learning method on datasets with highly homologous proteins, because conserved consensus patterns not necessarily related to the function of the proteins could be discovered instead of the sought patterns responsible for the function.

### PD138/NB843 Dataset

We performed an additional experiment involving the method of Nimrod et al. [4] on the dataset PD138/NB843. The method of Nimrod et al. exploits also evolutionary information therefore it is interesting to see whether methods relying only on physicochemical and/or structural features could come close to its predictive accuracy.

In this additional experiment, we used only random forest classifier because this classifier was also used by Nimrod et al. The AUC values of the approaches based on the physicochemical features (PF), structural features (SF), and their combination (PSF) were (respectively) 0.84, 0.82, and 0.82, whereas the method of Nimrod et al. achieved AUC of 0.9. This indicates that there is still a large gap between the structural and physicochemical feature based approaches on one hand, and methods relying on evolutionary conservation information.

### Evaluation of binding motif independence

In order to further support our claim that the patterns discovered for DNA-binding proteins in general (datasets PD138, UD54, BD54, APO104) did not just capture the consensus patterns of particular folds, we performed an experiment in which the relational learning model was always constructed for proteins from all but one protein group and then tested on this excluded group. Proteins of the dataset PD138 were divided into seven groups following the work of Szilágyi and Skolnick [8]. They were the following: helix-turn-helix, zinc-coordinating, zipper-type, other *α*-helix, *β*-sheet, other and enzyme. We used linear SVM based on our structural features (SF), because SVM turned out to perform best in the experiments described in Table 2. We show both the predictive accuracies obtained by testing the learnt classifiers on the excluded groups and the cross-validated accuracies obtained by the classifiers on the remaining parts of the dataset in Table 6. The resulting accuracies on the excluded groups, which should correlate with the ability of our method to discover patterns characteristic for DNA-binding proteins in general, are reasonably high with the exception of the enzyme group. This agrees with the results of Szilágyi and Skolnick [8] and Stawiski et al. [5], who also noticed a drop in the ability of their method to detect DNA-binding proteins in the enzyme group. We can conclude that our method is indeed able to construct classifiers which can work accurately over various (non-enzyme) groups of proteins and that its ability to detect DNA-binding proteins is not due to discovery of conserved consensus patterns of different protein folds.

**Table 6:** Evaluation of binding motif independence

**Protein group**	**Accuracy on**	**Cross-validated accuracy**
	**excluded group**	**on training data**
Helix-turn-helix	83.3	80.3
Zinc-coordinating	100	82.9
Zipper-type	88.9	83.1
Other *α*-helix	100	85.0
*β*-sheet	77.8	86.0
Other	100	82.5
Enzyme	58.1	90.4

Predictive accuracies obtained by linear SVM classifiers trained on the datasets PD138/NB110 with protein groups excluded from PD138. The accuracy on excluded group is the percentage of correctly classified proteins from the protein group excluded from the training data. The cross-validated accuracy on training data is the accuracy of the learnt model estimated by 10-fold cross-validation on the training data.

## Conclusions

We applied relational machine learning techniques to predict DNA-binding propensity of proteins. We utilized our relational learning method [11]. We have shown that our relational learning approach is competitive to a state-of-the-art physicochemical approach for DNA-binding propensity prediction in terms of predictive accuracy. Moreover, we have illustrated that our method is capable to also provide interpretable patterns describing spatial configurations of amino acids in protein structures. In the future we would like to apply the method to protein function prediction in general.

## Endnotes

^a^This definition is equivalent to what is known as hypergraph homomorphism or *θ*-subsumption in inductive logic programming [14].

^b^http://www.pdb.org

^c^Srinivasan, A.: The Aleph Manual, 4th edn. (2007), http://www.cs.ox.ac.uk/activities/machlearn/Aleph/aleph.html

^d^We are grateful to an anonymous reviewer of the paper for pointing out this fact.

## Competing interests

The authors have no competing interests to declare.

## Authors’ contributions

Fž and JT conceived the idea of using relational machine learning for DNA-binding prediction. AS and OK conceived, designed and implemented the method, performed the experiments and analysed the results. AS, OK, Fž and JT wrote the paper. All authors read and approved the manuscript.
